# Microsurgical Calcaneus Osteocutaneous Fillet Flap for Below-Knee Amputation Salvage: A Case-Based Surgical Technique with Long-Term Outcomes

**DOI:** 10.1007/s43465-025-01569-1

**Published:** 2025-09-23

**Authors:** Paolo Titolo, Giuseppe Di Palma, Alessandro Crosio, Mario Ronga, Davide Ciclamini

**Affiliations:** 1 Reconstructive Microsurgery Unit, Department of Orthopedics & Traumatology, AOU Città della Salute e della Scienza di Torino, 10126 Turin, Italy; 2 School of Medicine and Surgery, University of Turin, Turin, Italy; 3 Orthopaedic and Trauma Operative Unit, Department of Health Sciences, University of Eastern Piedmont, Novara, Italy

**Keywords:** Calcaneal fillet flap, Leg amputation, Lower limb open fracture, Leg prostheses, Leg stump, Foot fillet flap, Osteocutaneous fillet flap, Pulsed dose radiofrequency

## Abstract

Traumatic amputations and non-reconstructable sub-amputations with soft-tissue loss of the lower limb are highly disabling events. The calcaneus osteocutaneous fillet flap should be considered in lower leg amputations and non-reconstructable sub-amputations to preserve length and convert an above-knee amputation to a below-knee amputation without further donor site morbidity. Preserving the knee joint in an amputated limb is associated with faster rehabilitation and a quicker, more natural gait. Some complications can occur and must be appropriately managed during the postoperative days and the following months. This report presents a case of a fillet flap successfully performed in a young woman, with an extended 6-year follow-up. Three consecutive complications, i.e., osteosynthesis revision, painful saphenous nerve amputation neuroma, and surgical scar dehiscence, were successfully managed in the postoperative weeks. At 6 years from injury, the patient had a physiological gait and walked a distance of 200 m in 2 min without shortness of breath. She scored optimally (82 out of 100 points) in all the items of the Short-Form Health Survey SF-36 test (compared with the standard scores for below-knee amputated patients). She scored 63 out of 80 on the Orthotics and Prosthetics User's Survey (OPUS) test. No further revision surgery was necessary. No sensory disturbances persisted after the first year from the trauma. The patient reported occasional mood swings coinciding with episodes of phantom limb pain that continued throughout the years and needed painkillers.

## Introduction

In traumatic amputations or crush injuries of the lower limb, the high energy of the trauma often results in severe limb damage and proximal soft-tissue loss, which makes a standard"below the knee"amputation not amenable. In such a case, both amputation (above the knee) and replantation (if indicated) can be possible. Unfortunately, too often, the ischemia time, the presence of comorbidities, or the need for other life-saving procedures make amputation necessary. Non-reconstructable severe Gustilo 3 open fractures frequently require above-knee amputation. In such a case, preserving the length of the stump is essential, as a below-knee amputation (BKA) results in decreased rehabilitation time and improved function compared to an above-knee amputation (AKA) [1]. To perform a below-knee amputation, a well-planned bone resection at least 6 cm below the tibial tuberosity is necessary, but often the proximal stump is much shorter. Stable and good-quality soft-tissue coverage of the stump is required to guarantee optimal prosthetic fitting, which is often impossible because of proximal or around-the-knee soft-tissue losses. The"spare parts"concept, first described by Jupiter in 1982, Colen in 1983, and Russell [2] in 1986, can be applied to allow a BKA instead of an AKA in acute trauma cases [3]. Some predictable complications, not thoroughly described in the literature, may occur, and surgeons must be aware of and ready to address them properly. We report a case with a 6-year follow-up and a sequence of complications.

## Case Report

**Fig. 1:**
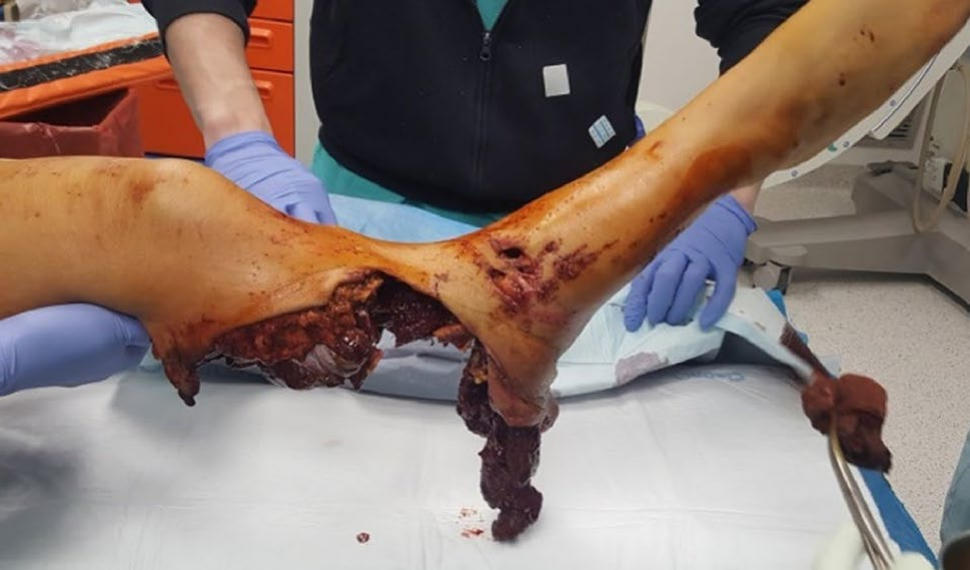
Proximal right leg sub-amputation at presentation in the emergency room

**Fig. 2:**
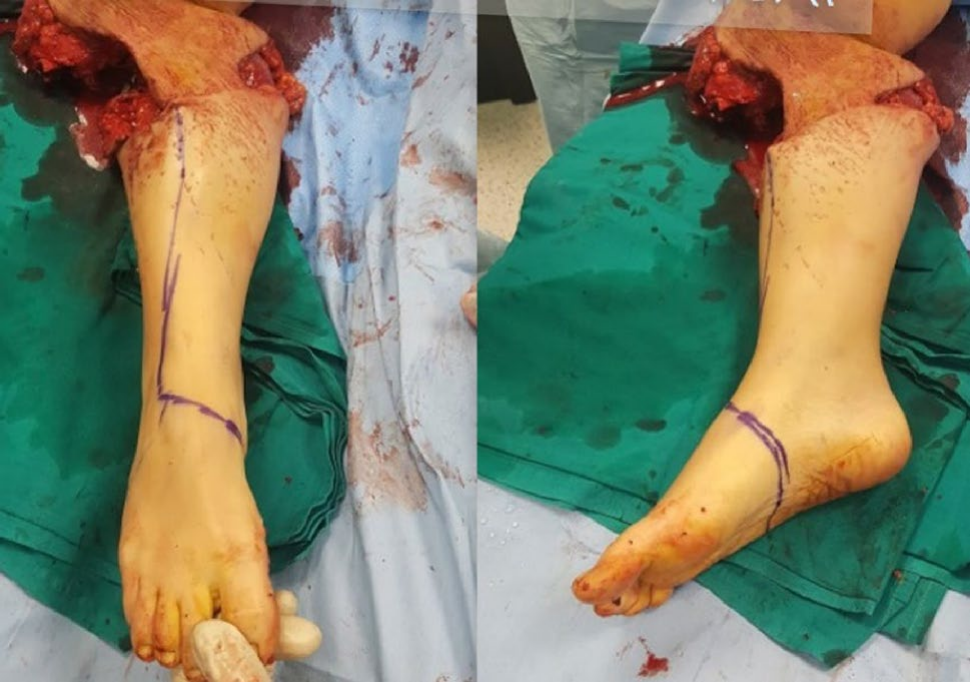
Drawing of the osteocutaneous calcaneal fillet flap on the sub-amputated leg before raising it

**Fig. 3:**
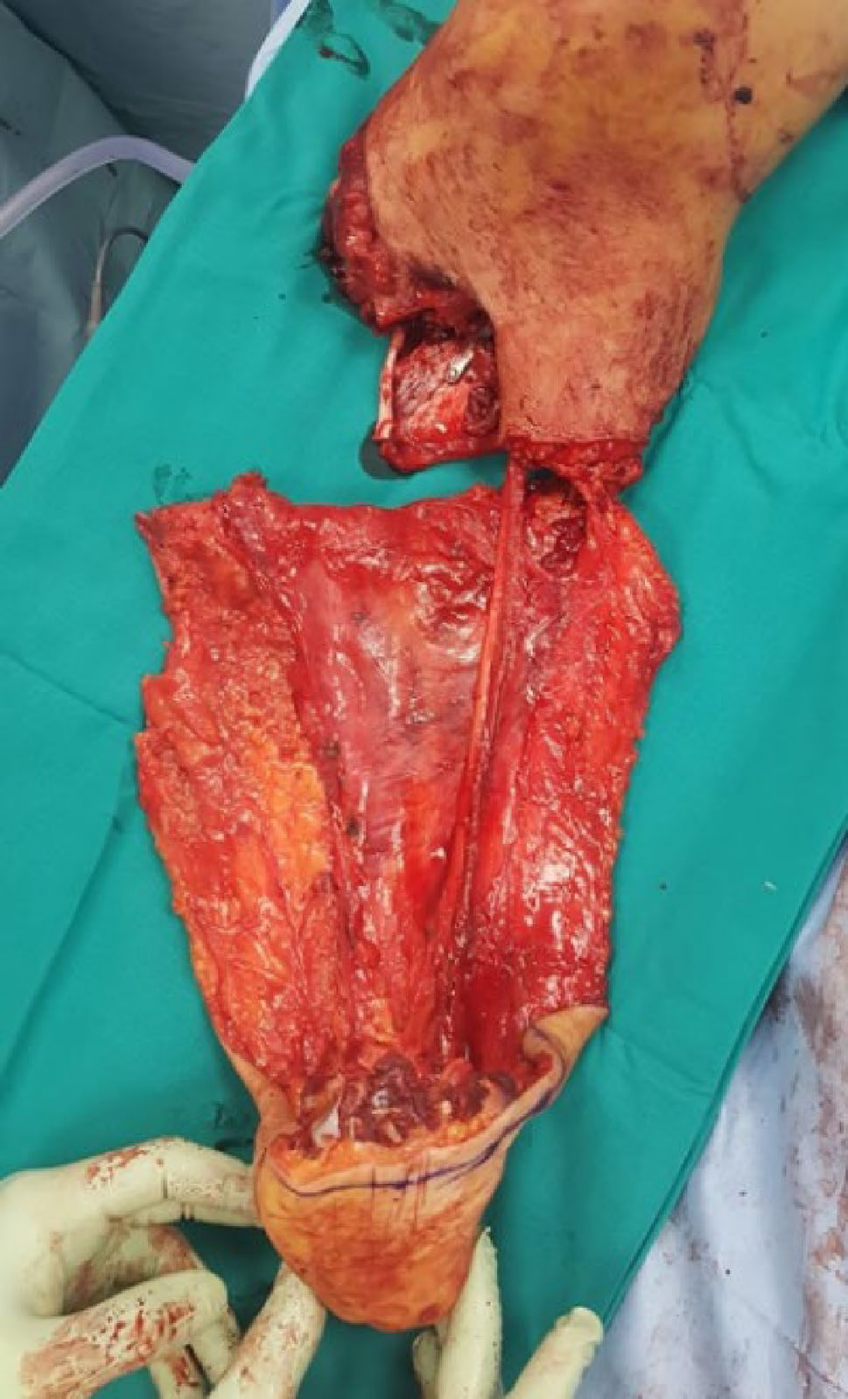
The raised osteocutaneous calcaneal fillet flap with posterior tibial nerve continuity before the insetting

**Fig. 4:**
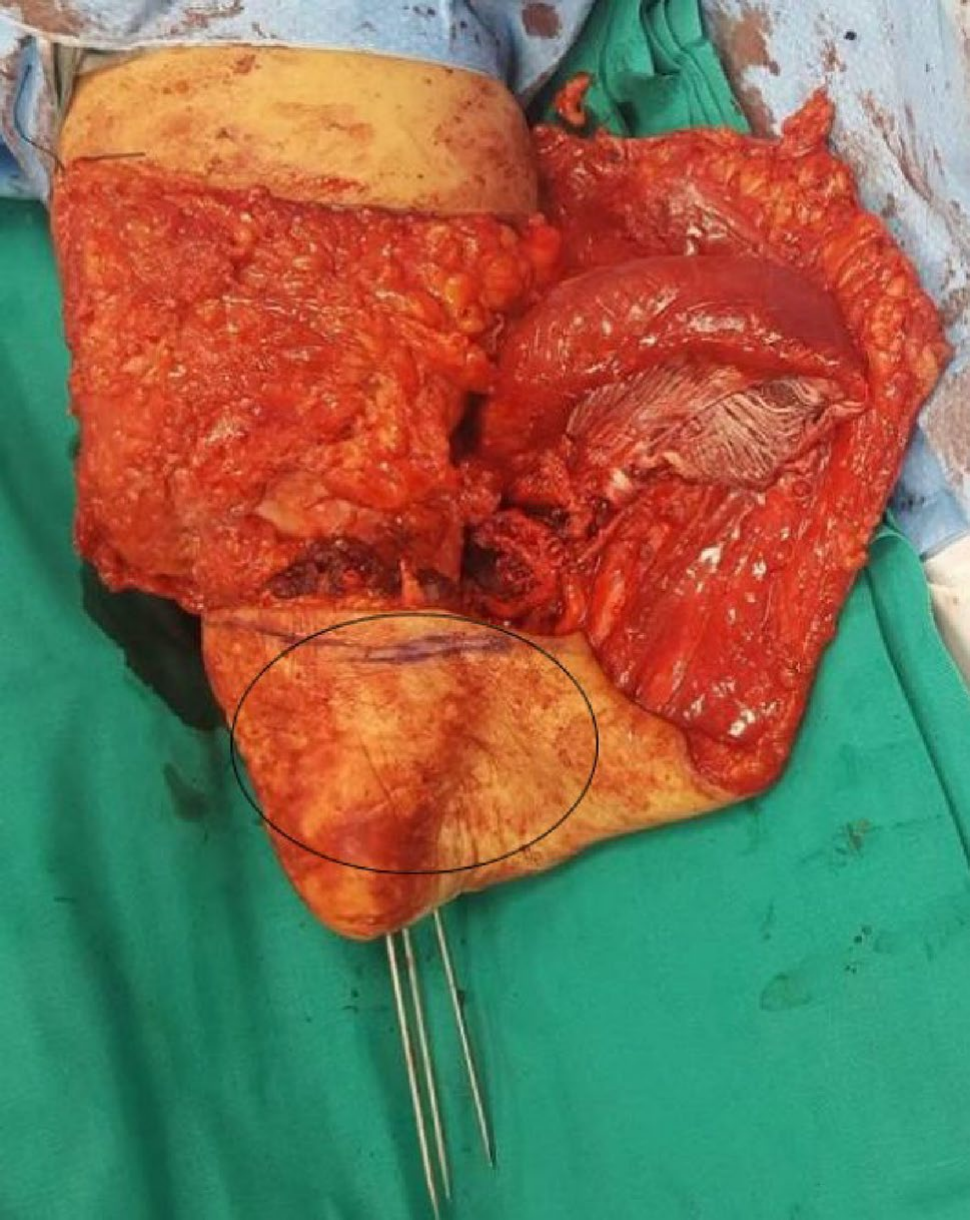
Right knee and leg: calcaneal fillet flap positioned in reverse mode and fixed with three Steinman Wires

**Fig. 5:**
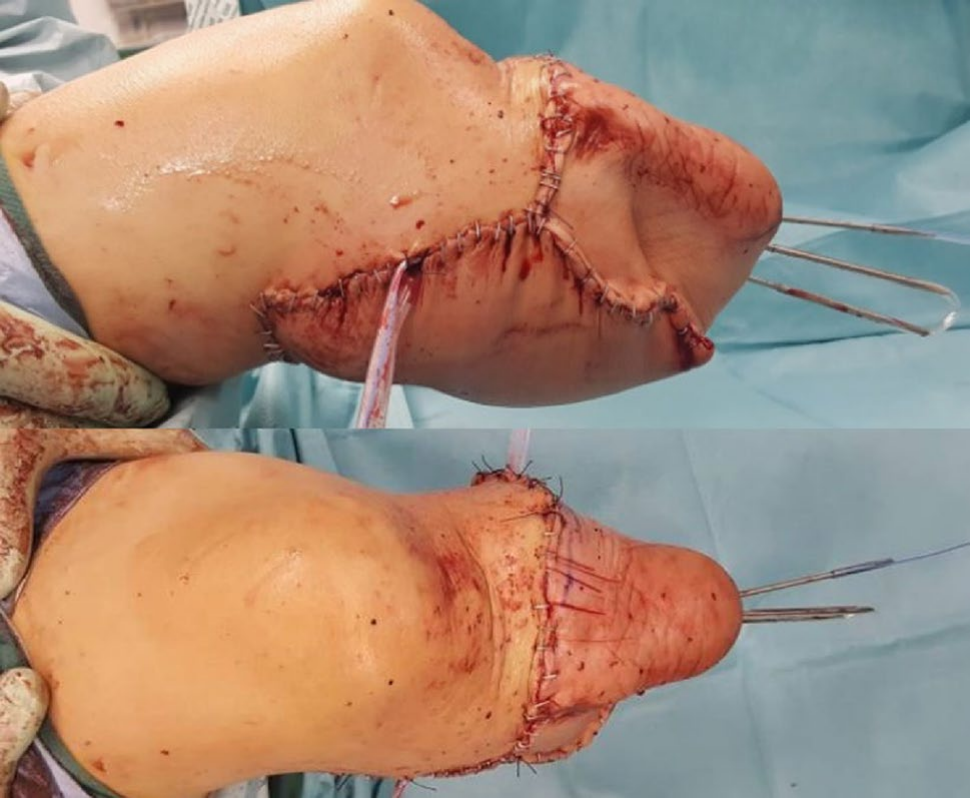
Right knee with calcaneal fillet flap in place and final skin closure: lateral view (upper image) and anterior view (lower image)

A young woman sustained a crush injury of the right lower limb with a sub-circumferential wound of the proximal leg (Fig. 1), complete muscle lesions, proximal leg soft-tissue loss, Gustilo IIIC open tibial and fibular fractures 2 cm below the anterior tibial tuberosity, and no arterial bleeding distal to the wound. The MESS score was 7. No other injuries were found. X-rays and CT scans were taken. In the O.R. examination, the skin was degloved from the posterior distal thigh to the middle third of the anterior leg. The tibial nerve was crushed but in continuity. Revascularisation with preservation of the lower leg was not recommended because of the severe muscle trauma and the prolonged ischemia time, which could put the patient's life at risk. The skin and soft-tissue degloving injury would also require a flap to cover the bone and any sort of osteosynthesis at the level of the fracture. In such a case, a too-short proximal tibia stump and the soft-tissue loss at the injury level and proximal to it make a BKA not feasible. To avoid an AKA, the surgeon proceeded with an osteocutaneous calcaneal fillet flap (Figs. 2, 3), as described by many authors. The tibia was cut 1 cm below the tubercle, and the proximal tibiofibular joint was stabilized. The tibial nerve was preserved and placed in soft tissue, and the heel was disarticulated while maintaining the skin and plantar muscles. The flap was then reversed and fixed with Steinmann wires (Fig. 4). End-to-end microsurgical anastomosis was performed between the popliteal artery, the posterior tibial artery and two concomitant veins. Eventually, skin closure without tension was feasible (Fig. 5).

**Fig. 6:**
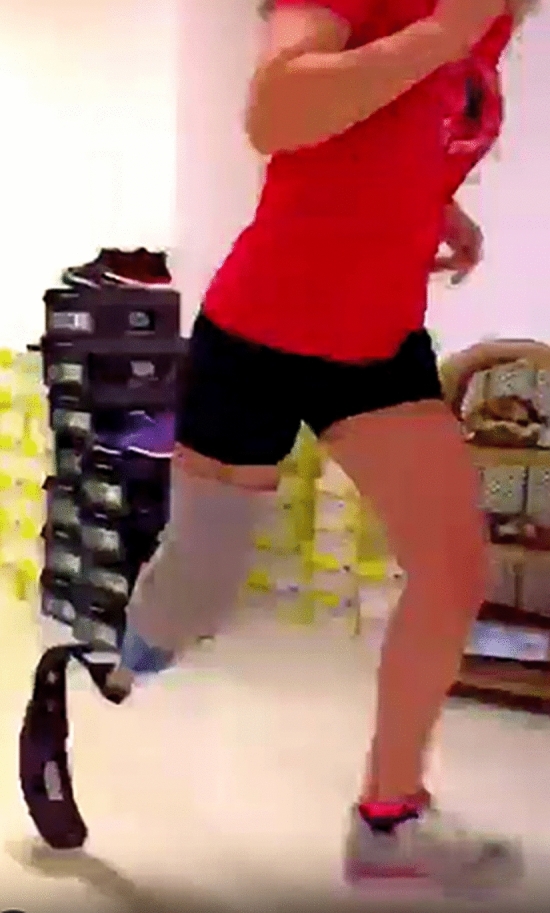
Normal gait 6 years after trauma

After surgery, anticoagulant (LMWH) and cardioaspirin (ASA) were administered. The patient started immediate antibiotic prophylaxis for open fractures and phantom limb pain prophylaxis with duloxetine and pregabalin. Early knee mobilization began in the next postoperative days. Nineteen days after the first surgery, to close the stump, to avoid the risk of local infection, and to get a more stable fixation, the surgeon revised the osteosynthesis (which was unstable), removing the Steinman wires and fixing the calcaneal bone with three 6 mm-diameter cannulated screws. Six weeks after the trauma, we used pregabalin and pulse-dose radiofrequency (PDRF) on the proximal stump of the saphenous nerve to treat hyperalgesia and allodynia. Eight weeks after the trauma, the patient was discharged. At the 4-month follow-up, wound dehiscence due to a superficial soft-tissue infection was treated with local debridement and antibiotic therapy until complete resolution. At 5 months post-op, a definitive prosthesis was placed. At the 1-year follow-up, the knee range of motion was almost full, and the patient had a physiological gait with the prosthesis. The patient has been on an annual follow-up, and 6 years after the initial operation, she can freely use her right leg with a well-fitting prosthesis (Fig. 6). No revision surgery was necessary. No sensory disturbances have been reported, though occasional mood swings have been noted, coinciding with episodes of phantom limb pain treated with painkillers. No ulcers have been reported. At the last visit, conducted at the end of 2024, the patient underwent a performance test, the 2-min walk test. The patient was instructed to walk as far as possible in the 2 min. She walked 202 m in 2 min without shortness of breath. We administered the 36-item Short-Form Health Survey (SF-36) questionnaire, which assesses the quality of life and rehabilitation success of people with lower limb amputations, and the Orthotics and Prosthetics User's Survey (OPUS) test, which measures outcomes relevant to the prosthetic and orthotic device user. She scored 82 out of 100 on the SF-36 and 63 out of 80 on the OPUS test.

## Discussion

Typically, to perform a BKA, 6 cm below the tibial tuberosity or 12.5–17.5 of tibial length from the distal to the medial tibial articular surface is required [4]. Preserving the knee joint in an amputated limb is associated with faster rehabilitation and a quicker, more natural gait. The osteocutaneous flap allows for an appropriate stump length for prosthetic fitting and provides a sensate weight-bearing tissue. Such a technique ensures better proprioception and reduces prosthesis-related complications. To achieve this, preserving the calcaneal tuberosity in the flap is essential to maintain the vertical fibrous structure between the calcaneal tuberosity and the deep fascia, which resists shearing forces caused by prosthetic use. This flap can be raised as a pedicled flap (in sub-amputation) or as a free flap in amputation or sub-amputation with blood supply damage [3]. In the case of vascularized feet, the choice of the fillet flap can be delayed and performed lately after thorough and even multiple debridements [5]. Sometimes, even in the case of a well-vascularized foot, damage to the main vessels can be present (like subclinical intimal trauma). Surgeons must be cautious to avoid subsequent ischemia or venous congestion in the case of pedicled fillet flap [5]. In a pedicled fillet flap, a modification in the free flap could be advised in case of vessel damage or severe vessel kinking due to shortening, especially for veins, since congestion is a common complication [1]. In our case, the posterior tibial artery was severed, and we needed a free flap technique in an emergency with vessel anastomosis. The flap required no revision of the anastomosis in the following days. The proximal tibial stump lengthening with free or pedicled fillet flap also presents some complications, such as procedure failure and revision into AKA, wound dehiscence, allodynia, hyperalgesia, skin ulcers and maceration, infection, non-union, stump bulkiness, phantom limb pain, and neuroma. Getting a stump free of pain is of utmost importance in this procedure. Prompt referral to a pain specialist or immediate treatment with specific medications is the key to the prevention of phantom limb and painful neuromas. Our patient developed severe pain on the proximal stump of the saphenous nerve with hyperalgesia and allodynia. We decided to treat this complication with pregabalin and pulsed dose radiofrequency (PDRF). Radiofrequency (RF) use in treating chronic pain has a long history, beginning in 1920. PDRF utilizes high-frequency electrical currents to create an electrical field, which does not cause an irreversible destruction of tissues [6]. An ultrasound-guided needle-shaped device reaches the nerve stump and delivers an energy dose directly to the nerve. PDRF machine waits for the temperature to come back under 42 °C to give the following impulse with the same characteristic. The electrical dose is always controlled and known, causing no damage. Some authors hypothesized that electric fields alter immune modulation: the magnetic field reduces the proinflammatory cytokines, such as interleukin (IL)−1b, tumor necrosis factor (TNF)-alpha, and IL-6 and IL-8 [7, 8]. After two treatments, the patient experienced prompt improvement and a definitive resolution of saphenous nerve stump pain without further surgery. Recurrent but unfrequent episodes of phantom limb pain persisted during the following 6 years (in accordance with lower mood swings), but they could be easily treated with mild painkillers.

The hospital stay length of our patient was 56 days, which included the management of the first two out of three complications (osteosynthesis revision and saphenous nerve stump neuroma PDRF treatment). Ghali S. et al. [5] report a mean hospital stay of 55.5 in their case series of 6 patients (UK, London). Xiao [9] interestingly reports a significantly shorter hospital stay of 20.4 days in their case series (Kenya), but the reduction of the hospital stay seems to be related to the elective three out of five reported cases. The duration of the hospital stay for patients with such severe trauma confirms the complexity of treating this kind of lesion and the need for further improvement in the field. Giving the correct surgical indication, keeping a timely schedule of consequent procedures, and promptly addressing frequent complications can reduce the length of stay, improve patient recovery, and reduce costs.

When comparing our results with the literature, a substantial correspondence with the mean outcomes and rate of complications arises. Painful neuromas seem to be uncommon, but they are described in traumatic setting [10]. To our knowledge, no report exists of any PDRF application in the case of free or pedicled fillet flap technique. This non-invasive technique could offer a valuable option to avoid more invasive surgical procedures (like surgical exeresis of the neuroma) with uncertain outcomes [6].

In conclusion, the free or pedicled osteocutaneous fillet flap is a well-established technique with fully described complications to convert an AKA to a BKA in emergency or delayed settings. As previously proved in the literature, the correct surgical indication and optimal complication management can generally get the amputated patient to achieve a pain-free gait while wearing a well-fitted prosthetic leg on a pain-free below-the-knee stump.

## Data Availability

The data underlying this case-based surgical technique report were utilized exclusively for the preparation of the manuscript. In accordance with patient confidentiality and privacy regulations, these data were not retained and are therefore not available for further sharing.
